# Role of the Plasticity-Associated Transcription Factor Zif268 in the Early Phase of Instrumental Learning

**DOI:** 10.1371/journal.pone.0081868

**Published:** 2014-01-23

**Authors:** Matthieu Maroteaux, Emmanuel Valjent, Sophie Longueville, Piotr Topilko, Jean-Antoine Girault, Denis Hervé

**Affiliations:** 1 Inserm UMR-S 839, Paris, France; 2 Université Pierre et Marie Curie, Paris, France; 3 Institut du Fer à Moulin, Paris, France; 4 Inserm UMR-S 661, Montpellier, France; 5 UMR 5203 CNRS, Montpellier, France; 6 Université Montpellier I & II, Montpellier, France; 7 IBENS, Developmental Biology Section, CNRS UMR 8197, Inserm UMR-S 1024, Ecole Normale Superieure, Paris, France; University of Chicago, United States of America

## Abstract

Gene transcription is essential for learning, but the precise role of transcription factors that control expression of many other genes in specific learning paradigms is yet poorly understood. Zif268 (Krox24/Egr-1) is a transcription factor and an immediate-early gene associated with memory consolidation and reconsolidation, and induced in the striatum after addictive drugs exposure. In contrast, very little is known about its physiological role at early stages of operant learning. We investigated the role of Zif268 in operant conditioning for food. Zif268 expression was increased in all regions of the dorsal striatum and nucleus accumbens in mice subjected to the first session of operant conditioning. In contrast, Zif268 increase in the dorsomedial caudate-putamen and nucleus accumbens core was not detected in yoked mice passively receiving the food reward. This indicates that Zif268 induction in these structures is linked to experiencing or learning contingency, but not to reward delivery. When the task was learned (5 sessions), Zif268 induction disappeared in the nucleus accumbens and decreased in the medial caudate-putamen, whereas it remained high in the lateral caudate-putamen, previously implicated in habit formation. In transgenic mice expressing green fluorescent protein (GFP) in the striatonigral neurons, Zif268 induction occured after the first training session in both GFP-positive and negative neurons indicating an enhanced Zif268 expression in both striatonigral and striatopallidal neurons. Mutant mice lacking Zif268 expression obtained less rewards, but displayed a normal discrimination between reinforced and non-reinforced targets, and an unaltered approach to food delivery box. In addition, their motivation to obtain food rewards, evaluated in a progressive ratio schedule, was blunted. In conclusion, Zif268 participates in the processes underlying performance and motivation to execute food-conditioned instrumental task.

## Introduction

Animals adapt their behavior when they receive reward, leading to incentive learning and changes in motivation state [1]. Learning processes are of two types, Pavlovian conditioning in which predictive associations of sensory stimuli with reward are memorized, and instrumental conditioning in which the consequences of motor acts are learned in relation with reward [2]. In combination with these learning processes, elements of stimulus-reward and action-reward associations acquire motivational values that greatly influence the animal willingness to perform the learned responses [3].

Considerable research has long suggested that these processes are controlled by the cerebral cortex and basal ganglia, the cortex being a major source of input to the basal ganglia through its topographical projections to the striatum [4]. In both rodents and humans, different types of conditioning are controlled by specific parts of the striatal complex that are connected with specific cortical areas [5], [6]. Despite some controversies in the literature, the ventral part of the striatum, or nucleus accumbens (NAc), appears to be more implicated in the acquisition and expression of appetitive Pavlovian responses whereas the dorsal striatum, or caudate-putamen (CPu), is preferentially involved in instrumental conditioning [7], [8]. In the CPu, the lateral part receiving innervation from the sensorimotor cortex has been distinguished from the medial part innervated by prefrontal cortex. Lateral CPu controls the formation of habits in which stimulus-response associations prevail with a loss of control by the outcome [9]. By contrast, medial CPu is critical for the formation of action-outcome associations mediating goal-directed behaviors [8].

Despite substantial progress in the identification of corticostriatal circuits involved in conditioning processes, little information is available about their molecular mechanisms. Dopamine plays a critical role in the consolidation of instrumental learning and Pavlovian conditioning through the combined action of dopamine D1 receptor (D1R) and glutamate NMDA receptors [10], [11], [12]. These results indicate that the coincident activation of D1R and NMDA receptors activates specific signaling pathways, promoting synaptic plasticity necessary for instrumental learning [13]. These mechanisms have many similarities with those required for the development of conditioned responses induced by psychostimulants, which also depend on co-activation of D1R and NMDA receptors [14]. Psychostimulant-induced conditioning activates the extracellular signal-regulated kinase (ERK) pathway through combined stimulation of D1R and NMDA receptors [15], [16]. Thus, the ERK pathway behaves as a logical “AND” gate, or coincidence detector, for plasticity [17]. Blockade of ERK activation inhibits both operant conditioning and psychostimulant-conditioned place preference [15], [18]. It is proposed that, during the learning sessions, ERK is activated in the striatum and engages transcriptional and translational mechanisms to enable corticostriatal plasticity and memory formation. However, the key proteins regulated by ERK during learning remain to be identified. Among the molecules downstream from ERK, Zif268, a transcription factor of the Egr family (Egr1, also known as Krox-24, NGFI-A, TIS8, AT225, G0S30, or ZNF225), is well characterized for its role in synaptic plasticity and memory consolidation [19]. Zif268 is strongly regulated by acute psychostimulant administration [20], [21] and participates in cocaine-conditioned place preference, a Pavlovian-type conditioning [22]. In the present study, we investigated whether Zif268 is induced during the first learning phases of a simple instrumental task. We determined the localization of this induction in the various areas of the striatum and the role played by contingency between operant behavior and reward. Finally, using Zif268-deficient mice, we studied the behavioral consequences of the lack of Zif268 on instrumental learning. Our results show that Zif268 is induced in the striatum by instrumental learning and is critical for the motivation to perform instrumental task.

## Materials and Methods

### Ethics statement

All the experiments were in accordance with the guidelines of the French Agriculture and Forestry Ministry for handling animals (decree 87–848). The laboratory was approved to carry out animal experiments by the Direction *Départementale des Services Vétérinaires de Paris, Service de la Protection et de la Sante Animales et de la Protection de l'Environnement* (licence B75-05-22). The experimental protocols were approved by the Institut du Fer à Moulin local review board. The principal investigators had a personal agreement (D Hervé, licence C-75-828; JA Girault, licence 75–877).

### Animals

Eight-week old C57BL/6J mice (Janvier, France) were used in the present study. They were maintained in 12 h light/dark cycle, in stable conditions of temperature (22°C) with freely available water. Food was freely available until 1 week before training. *Drd1a*::GFP mice were generated by the GENSAT (Gene Expression Nervous System Atlas) program at the Rockefeller University (New York, NY) on a Swiss Webster background [23]. These mice carried a recombined bacterial artificial chromosome (BAC) transgene expressing green fluorescent protein (GFP) under the promoter of the dopamine D1R gene. In these mice, GFP was shown to be expressed in less than 60% of medium-sized spiny neurons (MSNs), those containing D1R and projecting to the substantia nigra [24], [25], [26].

We used mutant mice with disrupted Zif268 gene in which a LacZ sequence was inserted 50 base pairs upstream of the start codon and a frameshift mutation was created [27]. Homozygous and heterozygous Zif268 mutant mice as well as their wild type littermates were produced from heterozygous mutant pairs that had been backcrossed on a C57BL/6N background for 10 generations. To compare their weight and food intake, homozygous (n = 7) and heterozygous (n = 7) mice as well as their wild type littermates (n = 10) were isolated in individual cages. After 9 days, the food intake per 24 h was evaluated by measuring the weight of pellets every day during five days (food ad libitum). Each individual was weighted the 14^th^ day.

### Instrumental learning protocol

One week before training, mice were food-deprived to adjust their weight to 90% of their initial weight. The weight was then maintained all along the experiments, by adjusting the food amount after each session, taking into account the food intake during the test. Mice were trained for instrumental operant learning using polymodal cages with two nose-poke holes on either side of a food delivery cup (Imétronic, France). In the “Active” group, mice had to poke in the “active hole” signaled by a white light above it, in order to get a 20 mg food pellet (Phymep, France) in the food cup. Poking in the “inactive hole”, with no light above, had no consequence. Following delivery of a pellet, the cue light was switched off for 10 seconds indicating no food was available during that period. In the “Yoked” group, mice were placed in the same boxes with two orifices (one indicated by a light and the other not). They received a food pellet when a paired “Active” mouse obtained one. In this group, the food delivery was thus not contingent on poking. Finally, in the “Control” group, mice were exposed to the same context, but no food was delivered when poking occurred in the signaled hole. During training, the numbers of pokes in the reinforced hole, non-reinforced hole, and food cup (full or empty) were recorded every 5 min, in addition to the number of obtained pellets. One animal of the Active group that did not obtain any food pellet at the end of the first training session was excluded from the analysis.

Homozygous (n = 11) and heterozygous (n = 16) Zif268 mutant mice and their wild type littermates (n = 18) were trained in 1-h daily sessions in the same operant boxes for a total of 15 days. The training was comprised of three session blocks of 5 days in which increasing fixed ratio (FR) schedules were applied: FR1 (days 1–5), FR5 (days 6–10) and FR10 (days 11–15). On the 16^th^ day, all the mice were tested on a 2-h progressive ratio (or PR) schedule, in which the number of pokes required for pellet delivery was increased after each delivery. The required numbers of pokes were increased as follows: 1, 2, 3, 5, 12, 18, 27, 40, 60, 90, 135, 200, 300, 450, 675, 1000, 1500, 2000, and 3000 (adapted from [28]). In this test, the breaking point was determined by the number of nose-pokes necessary for delivery of the last pellet obtained by the animals at the end of the session.

### Tissue processing for immunofluorescence and immunocytochemistry

Food-deprived mice were tested in a 1-h session of FR1 schedule for 1 or 5 days. After the last session, mice were rapidly anesthetized with pentobarbital (500 mg/kg, i.p.; Sanofi-Aventis) and perfused transcardially with 4% (weight/vol) paraformaldehyde in 0.1 M sodium phosphate buffer, pH 7.5. Brains were post-fixed overnight in the same solution and stored at 4°C. Thirty-micrometer thick coronal sections were cut with a vibratome (Leica) and stored at −20°C in a solution containing 30% ethylene glycol, 30% (both vol/vol) glycerol, and 0.1 M sodium phosphate buffer, until they were processed for immuno-labelling following the protocol described in [24]. After three 10-min washes in Tris-buffered saline (TBS; 0.10 M Tris and 0.14 M NaCl, pH 7.4), free floating brain sections were incubated for 5 min in TBS containing 3% H_2_O_2_ and 10% methanol, and rinsed again in TBS 3 times for 10 min. Brain sections were then incubated for 15 min in 0.2% (vol/vol) Triton X-100 in TBS, rinsed 3 times in TBS and blocked in 3% (weight/vol) bovine serum albumin–TBS solution before incubation overnight at 4°C with either rabbit monoclonal antibody directed against Zif268 (1∶1000, Cell Signaling Technology, ref. 15f7) or rabbit polyclonal antibody directed against c-Fos (1∶1000; Santa Cruz Biotechnology: ref. sc-52). The next day, brain sections were washed 3 times in TBS for 10 min and Zif268 or c-Fos immunolabelling was revealed by immunofluorescence in most experiments or by immunocytochemistry for some of them. For immunofluorescence, the sections were incubated with goat anti-rabbit Cy3-coupled antibody (1∶400; The Jackson Laboratories) for 45 min and, after two 10-min rinses in TBS and two 10-min rinses in Tris buffer (TB; 0.25 M Tris, pH 7.5), they were mounted in Vectashield (Vector Laboratories). For immunocytochemistry, after the primary antibody incubation and three rinses in TBS, the sections were incubated for 2 h at room temperature with the biotinylated secondary antibody (anti-IgG, 1∶200) in TBS. Sections were then incubated 2 h with avidin-biotin-peroxydase complex (ABC) solution (final dilution, 1∶50; Vector Laboratories, Petersbourgh, UK). After rinses in TB, sections were placed in a solution of TB containing 0.1% 3,3′-diaminobenzidine (DAB; 50 mg/100 ml) and developed by adding H_2_O_2_ (0.02%). They were mounted in Eukitt® (Sigma-Aldrich) after dehydration through alcohol and delipidation by xylene.

### Image acquisition and analysis of immunostained sections

Immunofluorescence was analyzed by sequential laser-scanning confocal microscopy (SP2; Leica) and peroxidase immunocytochemistry with a bright field microscope (DM6000B, Leica). Image analysis was performed at the *Institut du Fer à Moulin* Imaging facility. Simple or double-labeled pictures from the various regions of interest were taken in the caudate-putamen and nucleus accumbens at the 0.98 mm antero-posterior coordinate (distance from Bregma) according to the Paxinos and Franklin mouse atlas [29]. Neuronal quantification was performed in 375×375 µm images by counting cells above a fixed threshold using image-J software (http://rsbweb.nih.gov/ij/). For acquisition and cell counting, images were taken by an observer unaware of the treatment and randomized using home-made software before counting.

### Statistical Analysis

All statistical analyses were done using GraphPad Prism 5 software (GraphPad Software, Inc.) or Statistica (StatSoft, Inc). Numbers of Zif268 or c-Fos-positive cells and behavior parameters were analyzed using Student's t test or, one-way or two-way ANOVA followed by Bonferroni post-hoc tests. When appropriate, subjects were matched across sessions. Sphericity was tested using Mauchly's sphericity test and when assumption of sphericity was rejected, the results were adjusted using the Huynh-Feldt correction.

## Results

### Regulation of striatal Zif268 expression during operant conditioning in mice

**Figure 1:**
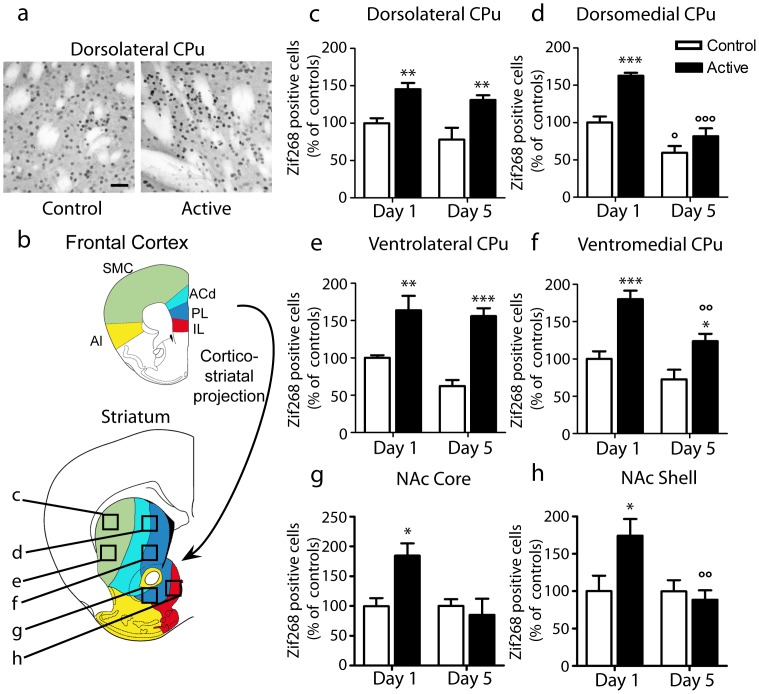
Zif268 protein expression after operant training. Zif268-positive cells were stained by immunocytochemistry after the 1^st^ or 5^th^ session of operant conditioning (FR1) in various areas of the striatum at the 0.98 mm antero-posterior coordinate [62]. a) Representative immunolabelling in the dorsolateral caudate-putamen (CPu) after the 1^st^ training session (**Active**) and in a control animal (**Control**). Scale bar: 50 µm. b) Positive cells were counted in the regions (375×375 µm) indicated with squares and the letters refer to the panels in which the results are shown. Schematic representation of the projection areas of the prefrontal cortex and sensorimotor cortices in the striatum (adapted from [30]). Quantification in the dorsolateral (c), dorsomedial (d), ventrolateral (e), ventromedial (f) CPu as well as in the core (g) and shell (h) of the nucleus accumbens (NAc), was performed by counting cells above a fixed threshold. Data were means ± SEM of 4 mice per group and analyzed using two-way ANOVA (Group x Session, see the F values in Results). Post-hoc comparison (Bonferroni test) of Active vs. Control: * p<0.05; ** p<0.01; *** p<0.001. Post hoc comparisons of Day1 vs. Day5: ° p<0.05, °° p<0.01; °°° p<0.001. ACd, anterior cingulate dorsal, AI, agranular insular, IL, infralimbic, PL, prelimbic, SMC, sensorimotor cortex.

To test if Zif268 expression could be altered by training for instrumental task, we subjected two groups of mice to a single or five sessions with a FR1 schedule (“Active”) and compared them to two control groups with 1 and 5 sessions, respectively (“Control”). The “Control” mice were exposed to identical chambers with the same environmental conditions, including the same lighting above one orifice, but nose-poking in the light-cued hole produced no delivery of food pellet. This excluded that Zif268 induction could be caused by some non-specific stimuli unrelated to the task. We measured the number of cells expressing Zif268 protein at the end of the session (1 h) using immunohistochemistry (Fig. 1a). We analyzed two regions receiving projections from the sensorimotor cortex: the dorsolateral (DL) and ventrolateral (VL) parts of the CPu; and four regions innervated by neurons originating in the prefrontal cortex: the dorsomedial (DM) and ventromedial (VM) CPu and the core and shell of NAc (Fig. 1b). We tested the effects of training (“Group” factor) and day of session (“Session” factor) on the results using two-way ANOVA. In almost all the tested areas of the striatum, this analysis indicated significant effect of Group factor, indicating differences between Active and Control animals (“Group” effect: F_(1,14)_ = 29.2, p<0.001; F_(1,14)_ = 26.2, p<0.001; F_(1,14)_ = 39.0, p<0.001; F_(1,14)_ = 34.5, p<0.001; F_(1,14)_ = 2.97, NS; F_(1,14)_ = 6.02, p<0.05, for DL-, DM-, VL- and VM-CPu, and core and shell of NAc, respectively). Post-hoc analysis showed significant increases in the number of Zif268-positive cells after the first training session in all these various striatal regions (Fig. 1c–h). After the 5^th^ training session, the activation of Zif268 transcription and translation, as evaluated by the number of Zif268-positive neurons, remained similar to that observed after the 1^st^ session in the DL- and VL-CPu (Fig. 1c,h), but was reduced in the other striatal regions (Fig. 1d, 1f–h). In the DL- and VL-CPu, two-way ANOVA indicated no significant effect of the “Session” factor (F_(1,14)_ = 4.04; F_(1,14)_ = 3.27, respectively) and no significant interaction between “Group” and “Session” factors (F_(1,14)_ = 0.177; F_(1,14)_ = 1.41, respectively). By contrast, for the DM-CPu as well as NAc core and shell, this analysis indicated a significant influence of “Session” factor (F_(1,14)_ = 53.8, p<0.001; F_(1,14)_ = 14.0; p<0.01; F_(1,14)_ = 7.54, p<0.05; F_(1,14)_ = 14.0; p<0.01, respectively), and significant interactions between “Group” and “Session” effects (F_(1,14)_ = 6.03, p<0.05; F_(1,14)_ = 5.75, p<0.05; F_(1,14)_ = 9.83, p<0.01, respectively), clearly showing that the activation of Zif268 gene was significantly different in the 1^st^ and 5^th^ session. After the 5^th^ session, in the DM-CPu and the NAc core and shell, the number of Zif268-positive neurons in the Active group was similar to that detected in the Control group (Fig. 1d, g, h). In the VM-CPu, the interaction between “Group” and “Session” factors was not significant (F_(1,14)_ = 1.70), although the “Session” factor was significant (F_(1,14)_ = 14.0, p<0.01). In consistency with these data, post-hoc analysis showed that the number of Zif268-positive neurons in VM-CPu was still significantly increased in the Active group after the 5^th^ session, but less than after the 1^st^ session (Fig. 1f).

Interestingly, the striatal regions in which Zif268 induction was reduced after the 5^th^ training session were those receiving projections from the prefrontal cortex [30] (Fig. 1b). In contrast, the DL- and VL-CPu, in which Zif268 activation was maintained after repeated training sessions, are innervated by the sensorimotor cortex.

### Zif268 induction required contingent association between operant behavior and reward delivery

**Figure 2:**
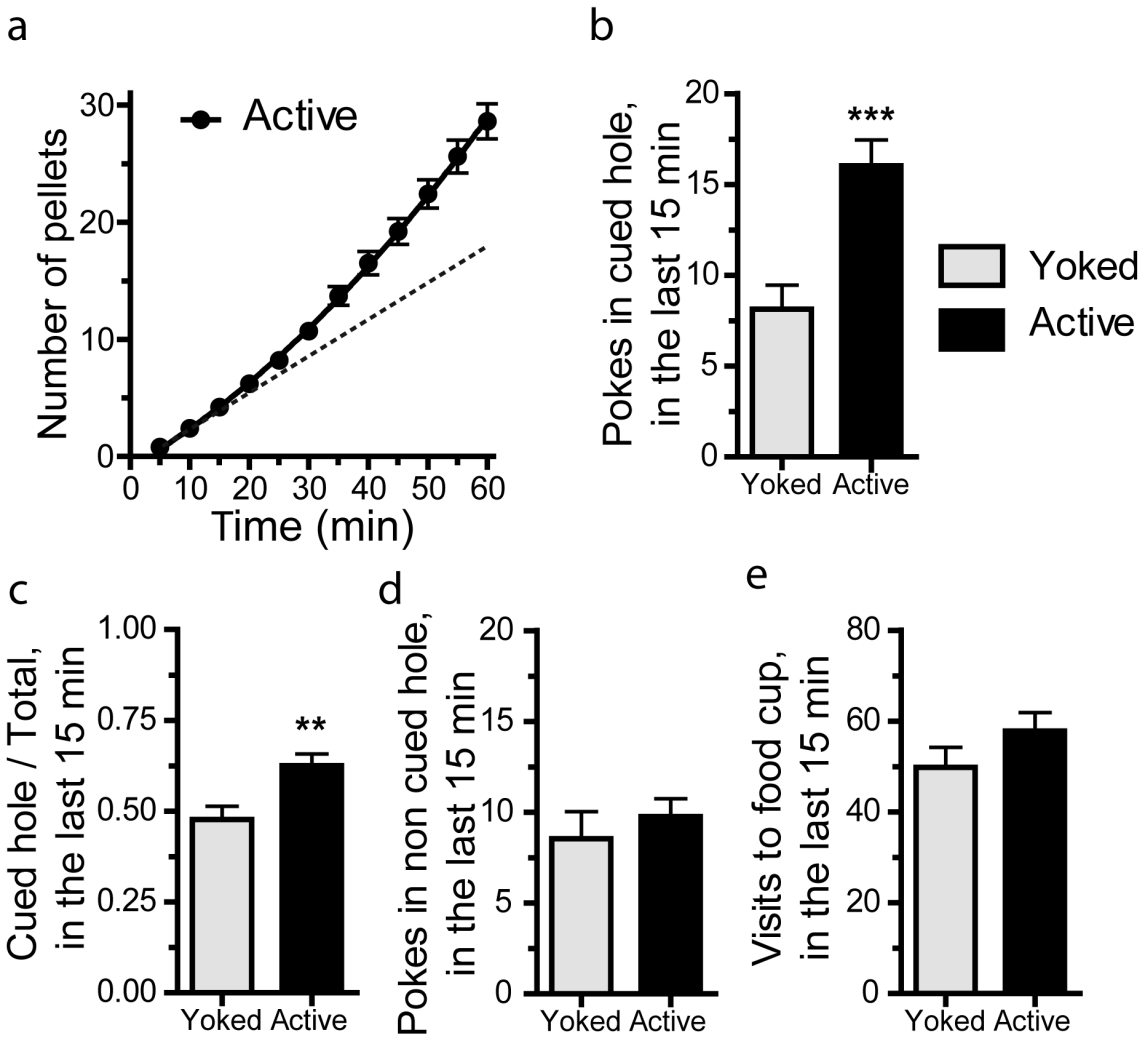
Mouse behavior during the first session of operant training. Behavioral parameters were measured in animals trained in 1-h session on FR1 schedule (**Active**, n = 84) and in yoked animals (**Yoked**, n = 46) receiving as many food rewards as Active animals but non-contingently. a) Cumulative number of pellets obtained by the Active group across the 1-h session. Dashed line corresponds to the curve slope at the beginning of session (between 5 and 10 min). b) Comparison of number of nose-pokes in the light-cued aperture in Active and Yoked mice during the last 15 min of 1-h session. In the Active group only, the nose-pokes into the light-cued hole provided food pellets. c) Ratio of nose-pokes in the light-cued hole and total nose-pokes in Active and Yoked mice during the last 15 min of 1-h session. d) Comparison of number of nose-pokes in the aperture not indicated by light in Active and Yoked mice during the last 15 min of the session. In both the Active and Yoked groups, poking in this orifice did not provide any reward. e) Comparison of number of visits to the empty food cup in Active and Yoked mice during the last 15 min of the session. Data (means ± SEM) were analyzed using Student's t test (see the t values in Results). ** p<0.01; *** p<0.001.

We then focused on the effects of the initial phase of training on Zif268 induction. To better analyze the parameters associated with Zif268 induction, we added a group of Yoked mice, which did not receive food pellets contingently to nose-poking but only when a paired animal of the Active group obtained rewards. We first compared the behavior of Active and Yoked groups. During the first training session, mice in the Active group displayed an increased success rate for gaining pellets after 30 min, showing their increased efficiency to perform the task (Fig. 2a). Acceleration of performance was associated with specific behavioral changes. When compared to the Yoked animals, the learning animals made more nose-pokes in the cued orifice during the last 15 min of the session (Fig. 2b; Student's t test: t_(128)_ = 3.68, p<0.001). During the same period, they displayed a higher ratio of pokes in the cued hole over total than the Yoked animals (Fig. 2c; t_(128)_ = 2.94, p<0.01), showing that they began to discriminate between reinforced and non-reinforced holes. In contrast, the number of nose-pokes in the non-cued orifice (non reinforced in the Active group) or visits to the empty food cup was similar in the two groups (Fig. 2d and e; t_(128)_ = 0.702, NS, and t_(128)_ = 1.25, NS, respectively). These behavioral differences between the two groups of animals clearly indicated that Active mice experienced and/or learned contingency at the end of the session. The use of the Yoked group allowed us to distinguish between the effects of food reward from those of operant learning per se.

**Figure 3:**
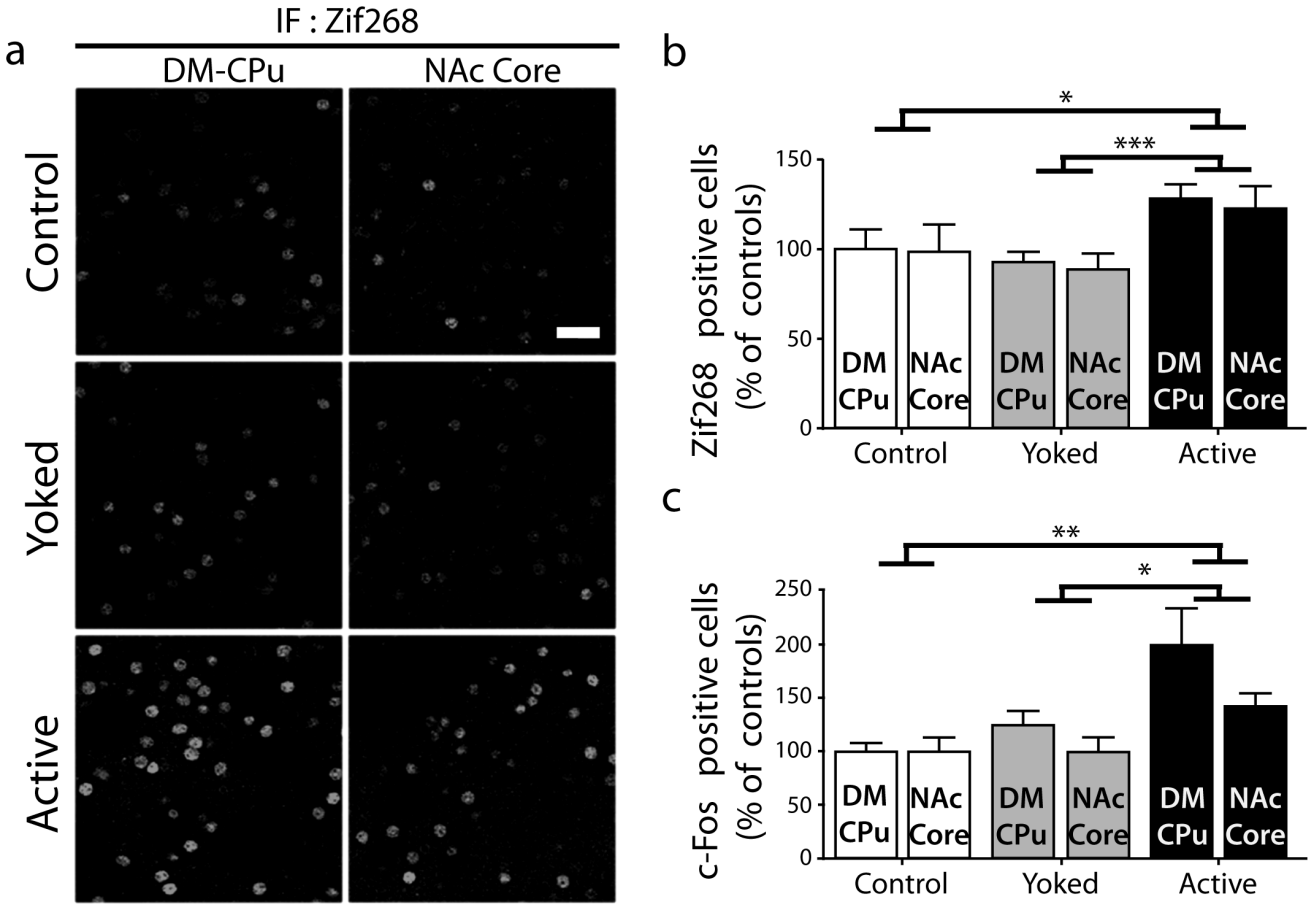
Zif268 and c-Fos protein expression after the first session of operant training. a) Zif268 immunofluorescence in the dorsomedial caudate-putamen (DM-CPu) and core of nucleus accumbens (NAc) in animals trained in FR1 schedule session (**Active**, n = 45), Yoked animals (**Yoked**, n = 42), and animals placed in the behavioral apparatus without food reward (**Control**, n = 23). Scale bar: 30 µm. Neurons immunoreactive for Zif268 (b) or c-Fos (c) were quantified on 375×375 µm confocal images of the DM-CPu and NAc core. Data (means ± SEM) were analyzed using two-way ANOVA (Group x Brain Structure, see the F values in Results). Post hoc comparison (Bonferroni test): * p<0.05; ** p<0.01; *** p<0.001.

We then compared in the Active, Control, and Yoked groups the number of Zif268-positive neurons in the DM-CPu and NAc core, two striatal regions innervated by the prefrontal cortex (Fig. 3a, b). Two-way ANOVA indicated a significant “Group” effect (F_(2,190)_ = 8.36, p<0.001) whereas the “Brain Structure” effect and the interaction were not significant (F_(1,190)_ = 0.023 and F_(2,190)_ = 0.023, respectively). In the absence of “Brain Structure” or interaction effects, we compared the Active, Control and Yoked groups of mice using Bonferroni post-hoc test. The number of Zif268-positive neurons was similar in the Yoked group and in the Control group receiving no reward. In contrast, this number was significantly higher in the Active group than in the Yoked (p<0.001) or Control (p<0.05) groups. Altogether, these results showed that the increased levels of Zif268 expression in the “Active” group was more associated with contingency (experiencing or learning) than with receiving rewards (Fig. 3a, b).

Very similar results were obtained when we examined the number of c-Fos-positive neurons in the DM-CPu and NAc core of the various groups (Fig. 3c). The two-way ANOVA indicated a significant “Group” effect (F_(2,104)_ = 5.24, p<0.01) but no significant “Brain Structure” effect (F_(1,104)_ = 1.09) nor interaction between the two factors (F_(2,104)_ = 0.41). The Bonferroni post-hoc tests used for evaluating the significance of differences between groups of animals indicated that the number of c-Fos-expressing neurons in the Active group was significantly higher than those in the Yoked (p<0.05) or Control (p<0.01) groups. In contrast, there was no significant variation in c-Fos expression between the Yoked mice and the animals receiving no reward.

In conclusion, Zif268 and c-Fos expression was increased in the DM-CPu or NAc core in mice after their first training session of operant conditioning. The lack of difference between mice just exposed to the operant chambers and those getting food pellets non contingently, clearly showed that gene induction was not associated with passively receiving food reward.

### Identification of the neurons in which Zif268 is induced during the initial operant training

**Figure 4:**
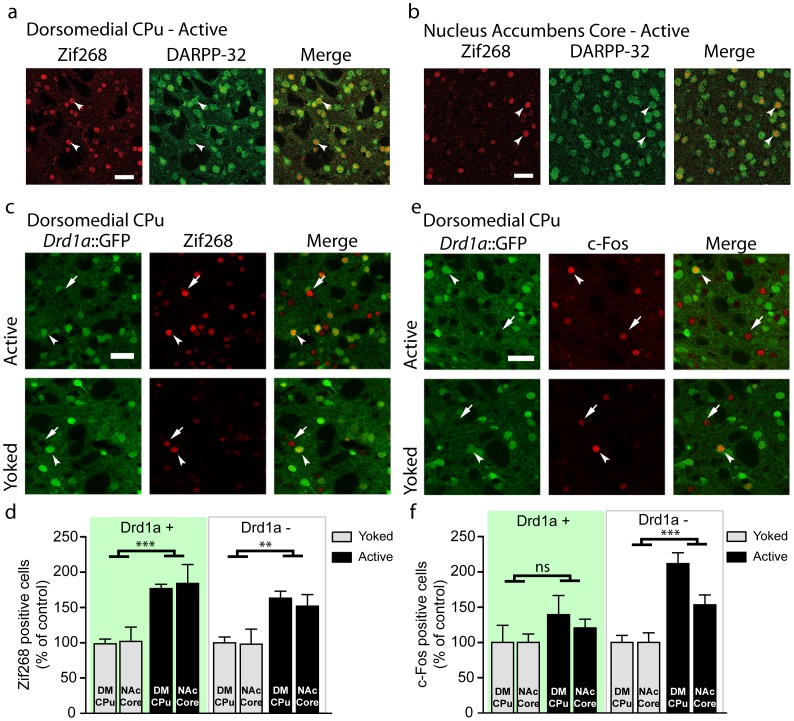
Zif268 is induced in D1 and D2 receptor-expressing MSNs after a single instrumental learning session in *Drd1a*::GFP transgenic mice. Colocalisation of Zif268 (red) and DARPP-32 (green) immunolabelling in the same MSNs in the dorsomedial caudate-putamen (CPu)(a) and nucleus accumbens core (b) after a single FR1 training session (Active mice). Arrowheads indicate co-expression of Zif268 and DARPP-32 in the same neurons. Scale bar: 40 µm. Confocal images of Zif268 (c) and c-Fos (e) immunolabelling (red) and D1 receptor promoter driven GFP fluorescence (green) in the dorsomedial caudate-putamen (DM-CPu) of Active and Yoked mice. Arrowheads indicate colocalization of immunolabelling and GFP expression; Arrows indicate absence of colocalization of immunolabelling and GFP expression. Scale bar: 40 µm. Number of Zif268 (b) and c-Fos (d) immunolabeled cells in GFP-positive (Drd1a +) and GFP-negative (Drd1a −) neurons in the DM-CPu and core of nucleus accumbens (NAc) of Active (n = 6) and Yoked (n = 6) mice. All the values are means ± SEM and the data are analyzed using factorial ANOVA (Group x Brain Structure x GFP Expression, see the various F values in Results). Post hoc comparison (Bonferroni test): ** p<0.01; *** p<0.001 Active vs. Yoked.

Following the first training session (“Active” animals), the vast majority of Zif268-positive neurons in the DM-CPu and NAc core were MSNs. Figs. 4a and 4b show double immunolabelling of Zif268 and DARPP-32, a MSN-specific marker. In 6 “Active” animals, 345 of 352 (97%) and, 251 of 264 (95%) Zif268-positive neurons were found to express significant amounts of DARPP-32 in the DM-CPu and NAc core, respectively. This analysis indicated that almost all the Zif268-positive neurons were MSN, but did not exclude the expression of Zif268 in the other types of striatal neurons, the aspiny interneurons which represent about 5% of striatal neurons in the rodent [31]. The MSNs are efferent GABAergic neurons comprising two sub-populations of roughly equal size that are distinguished on the basis of their projections and expression of specific genes [32], [33]. The striatonigral neurons project to the substantia nigra and the medial globus pallidus, and express D1R, whereas the striatopallidal MSNs terminate in the lateral globus pallidus and contain dopamine D2 receptors (D2Rs). To identify the MSNs in which Zif268 was induced following training to operant task, we took advantage of the *Drd1a*::GFP transgenic mice, which express GFP under the control of the D1R gene (*Drd1a*) promoter, hence tagging the striatonigral MSNs [23], [34]. The number of Zif268-positive GFP-positive and negative neurons in the DM-CPu and NAc core was compared in Active and Yoked *Drd1a*::GFP transgenic mice (Fig. 4a, b). The results were analyzed using factorial ANOVA (Group x Brain Structure x GFP Expression) and showed a very significant “Group” effect (F_(1,36)_ = 42.5, p<0.001) but no significant “Brain Structure” (F_(1,36)_ = 0.003) nor “GFP expression” (F_(1,36)_ = 1.25) effect. The various interactions between the three factors were not significant either (Group x Brain Structure, F_(1,36)_ = 0.018; Group x GFP Expression, F_(1,36)_ = 0.319; Brain Structure x GFP Expression, F_(1,36)_ = 0.327, Group x Brain Structure x GFP Expression, F_(1,36)_ = 0.093). Since we did not detect any significant difference between DM-CPu and NAc core, we used the Bonferroni post-hoc test to evaluate the significance of the Group effect. Zif268 expression was significantly increased in both GFP-positive and negative neurons (Fig. 4a, b). Previous studies have shown that GFP-negative MSNs in the *Drd1a::*GFP transgenic mice express D2R [26], therefore these results indicated that training to operant task induced Zif268 in both D1R- and D2R-expressing MSNs populations.

We also analyzed the number of c-Fos-positive neurons in the same animals in the GFP-positive and negative neurons (Fig. 4c,d). The factorial ANOVA (Group x Brain Structure x GFP Expression) of the results indicated significant effects of “Group” (F_(1,40)_ = 21.6, p<0.001) and “GFP Expression” (F_(1,40)_ = 4.69, p<0.05) factors as well as significant effect of Group x GFP Expression interaction (F_(1,40)_ = 4.69, p<0.05). In contrast, the “Brain Structure” effect was not significant (F_(1,40)_ = 2.47) as well as its various interactions with the other factors (Group x Brain Structure, F_(1,40)_ = 2.51; GFP Expression x Brain Structure, F_(1,40_ =  = 0.65; Group x Brain Structure x GFP expression, F_(1,40)_ = 0.65). Since the ANOVA indicated no significant difference between DM-CPu and NAc core, we evaluated using Bonferroni post-hoc test the significance of (Group x GFP Expression) effect. We found that c-Fos expression in learning animals was significantly increased in the population of GFP-negative MSNs expressing D2R (Fig. 4c, d). In contrast, the slight increase in c-Fos expression during the learning session in the GFP-positive MSN expressing D1R did not reach significance (Fig. 4c, d). In conclusion, the induction of Zif268 following initial training to operant conditioning occured in both D1R- and D2R-neurons whereas that of c-Fos took place in the D2R-neurons.

### Disruption of the Zif268 gene alters the acquisition of an operant task

**Figure 5:**
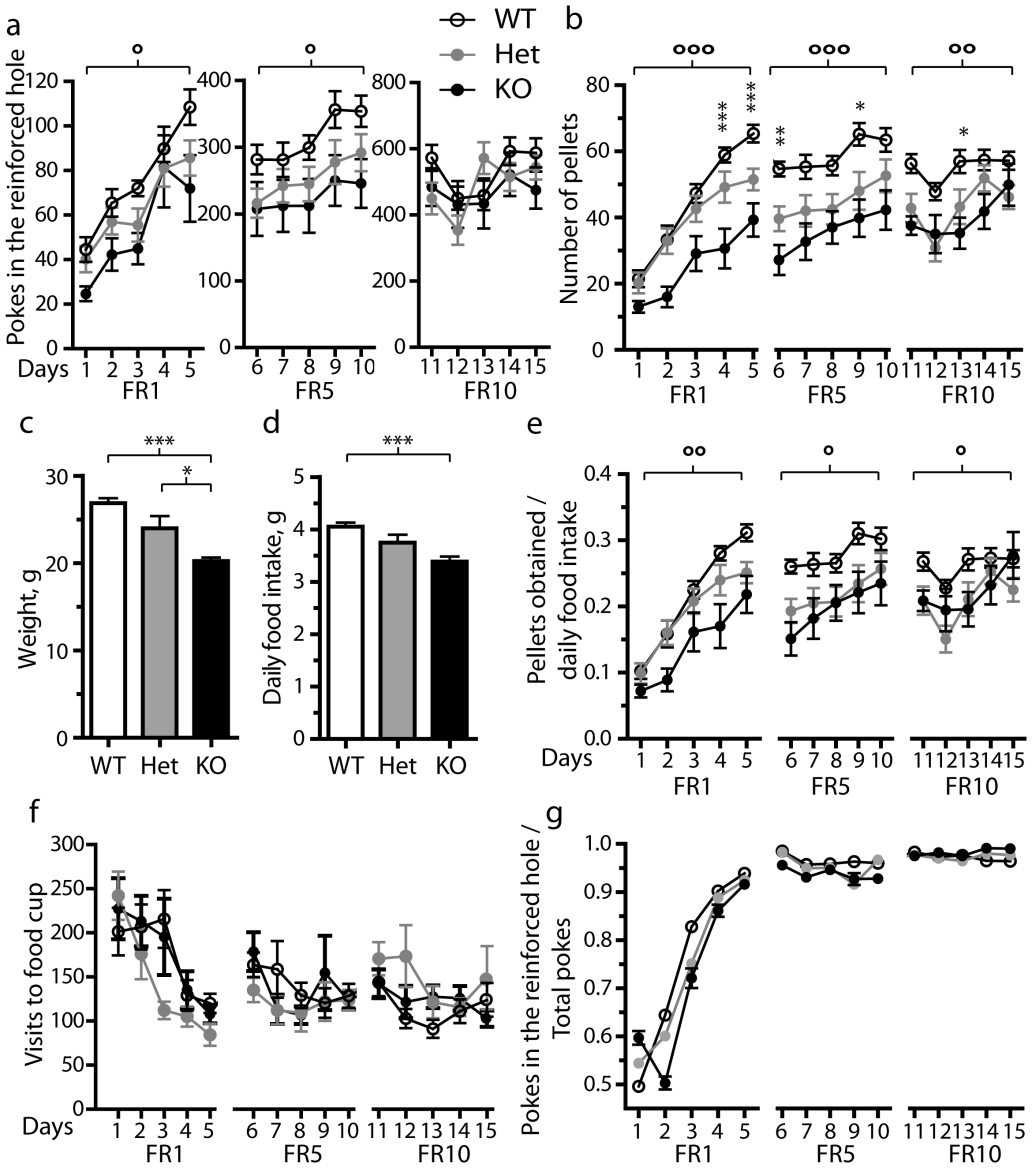
Operant behavior in Zif268 mutant mice under FR1, FR5 and FR10 schedules. Experiments were carried out in homozygous (**KO**) and heterozygous (**Het**) Zif268 mutant mice and their wild type (**WT**) littermates. a) Number of nose-pokes in the reinforced target across daily sessions with FR1, FR5 and FR10 schedule training. Homozygous mutant mice perform less nose-poking under FR1 and FR5 schedules than wild type but perform similarly under FR10. b) Pellets obtained across daily sessions with FR1, FR5 and FR10 schedule training. Zif268 mutation significantly reduced the number of rewards earned in the three blocks of training sessions. Weight (c) and daily food intake (d) of groups of KO, Het and WT mice. e) The amount of rewards obtained by the KO, Het and WT mice in 1-h session is evaluated by normalizing the number of gained pellets by the daily food intake in the three genotypes. Using this parameter, Zif268 mutation significantly alters the mouse performance in the three blocks of training sessions. f) The number of visits to the empty food cup is similar in the various genotypes. g) The ratio of reinforced and total nose-pokes is not significantly different in KO, Het and WT mice in the various training sessions. In a), b), e), f) and g), the values are means ± SEM (KO, n = 11; Het, n = 16; WT, n = 18) and the data are analyzed using repeated-measures two-way ANOVA (within-subjects factor of Session and between-subjects factor of Genotype; see the various F values in Results). Post hoc comparison (Bonferroni test): * p<0.05; ** p<0.01; *** p<0.001, homozygous vs. wild type. In a), b) and e), overall two-way ANOVA results for Genotype factor are indicated by: ° p<0.05; °° p<0.01, °°° p<0.001. In c) and d) the values are means ± SEM (KO, n = 7; Het, n = 7; WT, n = 10) and the data are analyzed using one-way ANOVA (see the various F values in Results). Post hoc comparison (Bonferroni test): * p<0.05; *** p<0.001.

Since Zif268 was induced as early as the first training session of instrumental task learning, we investigated whether its absence affected the acquisition of the task, and thereby characterized the role of this protein in the development of operant conditioning. To address this issue, we analyzed instrumental learning in Zif268 mutant mice with one or two null alleles of Zif268 gene [27]. The homozygous and heterozygous Zif268 mutant mice, as well as their wild type littermates, were trained to a 1-h session on FR1 schedule for 5 days. The fixed ratio was then increased to FR5 for another 5 days and finally to FR10 for 5 days. The day after the last FR10, the mice were subjected to a 2-h progressive ratio session. In order to follow the performance throughout the FR1, FR5 and FR10 sessions, the numbers of pokes in the reinforced hole were first analyzed by repeated-measures two-way ANOVA (within-subjects factor of “Session” and between-subjects factor of “Genotype”. FR1: Session, F_(4,168)_ = 39.6, p<0.001; Genotype, F_(2,42)_ = 4.15, p<0.05; interaction, F_(8,168)_ = 1.01, NS. FR5: Session, F_(4,168)_ = 6.66, p<0.001; Genotype, F_(2,42)_ = 3.92, p<0.05; interaction: F_(8,168)_ = 0.320, NS. FR10: Session: F_(4,168)_ = 7.08, p<0.01; Genotype, F_(2,42)_ = 2.46, NS; interaction, F_(8,168)_ = 1.08, NS). This analysis indicated significant effects of “Session” factor, implying that the mice commonly learned the task throughout the FR1, FR5 and FR10 sessions. ANOVA also showed that the “Genotype” factor was significant for the FR1 and FR5 sessions but not the FR10 sessions, the interaction between the Session and Genotype factors being never significant. Bonferroni post-hoc tests pointed out that the numbers of pokes in the reinforced hole were reduced in homozygous mutant mice as compared to wild type controls in the FR1 and FR5 (p<0.05) sessions but not in the FR10 sessions (Fig. 5a). This suggested that repetition of training sessions could compensate for the initial deficits seen in homozygous mutant mice. Interestingly, the scores of heterozygous mice were between those of homozygous and wild type mice. Post-hoc tests did not indicate any significant difference between the heterozygous and wild type mice nor between heterozygous and homozygous mice.

We then analyzed the number of rewards obtained by the various mice (Fig. 5b) using repeated-measures two-way ANOVA (FR1: Session, F_(4,168)_ = 83.4, p<0.001; Genotype, F_(2,42)_ = 10.8, p<0.001; interaction, F_(8,168)_ = 2.84, p<0.01; FR5: Session, F_(4,168)_ = 13.8, p<0.001; Genotype, F_(2,42)_ = 9.20, p<0.001; interaction: F_(8,168)_ = 0.670, NS. FR10: Session: F_(4,168)_ = 10.3, p<0.001; Genotype: F_(2,42)_ = 6.87, p<0.01; interaction: F_(8,168)_ = 1.81, NS). This analysis showed a significant effect of “Genotype” factor throughout the FR1, FR5 and FR10 sessions. More precisely, the Bonferroni post-hoc test indicated that the number of rewards obtained by the homozygous mutant mice was significantly lower than that obtained by the wild type mice all along the training sessions (FR1, p<0.001; FR5, p<0.001; FR10, p<0.01). However, the deficits tended to diminish when the training progressed. Again the performance of heterozygous mutant mice appeared intermediate between those of homozygous mutant and wild type mice (Heterozygous vs. Wild Type, FR5 sessions, p<0.05, FR10 sessions, p<0.05, Bonferroni post-hoc test).

However, the homozygous mutant mice were reported to display a lower weight than the wild type [27], suggesting a general deficit in food intake in these mice. We thus compared the weight of homozygous, heterozygous and wild type mice (Fig. 5c, one-way ANOVA, F_(2,23)_ = 16.5, p<0.001) and their daily food intake (Fig. 5d, one-way ANOVA, F_(2,23)_ = 9.95, p<0.001). Bonferroni test clearly indicated that the daily food intake of homozygous mutant mice was reduced as compared to that of wild littermates (p<0.001). The food intake was also reduced in heterozygous mutant mice but the difference failed to reach the significance threshold. To take into account the lower food intake in the mutant mice, the quantity of food received during the operant training was normalized to the daily food eaten by the respective genotypes (Fig. 5e). The data using this parameter for evaluating the amount of reward earned by the animals were analyzed with repeated-measures two-way ANOVA (FR1: Session: F_(4,168)_ = 82.6, p<0.001; Genotype: F_(2,42)_ = 5.93, p<0.01; interaction: F_(8,168)_ = 2.03, NS. FR5: Session: F_(4,168)_ = 14.2, p<0.001; Genotype: F_(2,42)_ = 5.05, p<0.05; interaction: F_(8,168)_ = 0.788, NS. FR10: Session: F_(4,168)_ = 10.3, p<0.001; Genotype: F_(2,42)_ = 3.48, p<0.05; interaction: F_(8,168)_ = 1.90, NS). This analysis clearly showed that the Genotype factor had significant effects in the FR1, FR5 and FR10 sessions. In addition, Bonferroni post-hoc tests indicated that the scores of homozygous mutant mice remained significantly lower than those of wild type controls during the FR1 and FR5 sessions (p<0.01 and p<0.05, respectively) but not during the FR10 sessions. The scores of heterozygous mice were also reduced (for FR10 sessions, p<0.05, Bonferroni post-hoc test). Therefore the lower daily intake of food in the mutant mice did not appear to be responsible for the difference in pellet earning.

In contrast with these alterations, homozygous, heterozygous, and wild type mice displayed similar numbers of entries into the food receptacle in the absence of food delivery (Fig. 5f). When repeated-measures two-way ANOVA was performed (FR1: Session, F_(4,168)_ = 13.8, p<0.001; Genotype, F_(2,42)_ = 1.02, NS; interaction, F_(8,168)_ = 1.17, NS. FR5: Session: F_(4,168)_ = 3.17, p<0.05; Genotype, F_(2,42)_ = 0.56, NS; interaction, F_(8,168)_ = 1.34, NS. FR10: Session, F_(4,168)_ = 4.36, p<0.01; Genotype, F_(2,42)_ = 0.41, NS; interaction, F_(8,168)_ = 1.86, NS), the Genotype factor never displayed any significant effect. The entries into the food receptacle were frequent in the first training days and became more sporadic thereafter. Together with their normal locomotor activity [22], these data suggested that the mutant mice did not display a general weakness, hypoactivity or hypoexploration that could have impeded their ability to perform nose-pokes. Another hypothesis that could explain their low performance in the instrumental task is a defect in learning capability, since homozygous Zif268 mutant mice display learning deficits in memory tasks [35]. To test this hypothesis, the ratio of nose-pokes in the active hole over total was analyzed with repeated-measures two-way ANOVA (Fig. 5d; FR1: Session: F_(4,168)_ = 78.8, p<0.001; Genotype, F_(2,42)_ = 1.07, NS; interaction, F_(8,168)_ = 1.87, NS. FR5: Session, F_(4,168)_ = 3.49, p<0.01; Genotype: F_(2,42)_ = 1.80, NS; interaction, F_(8,168)_ = 1.18, NS. FR10: Session, F_(4,168)_ = 0.46, NS; Genotype, F_(2,42)_ = 0.40, NS; interaction, F_(8,168)_ = 1.69, NS). The absence of significant effect of Genotype factor indicated that homozygous, heterozygous and wild type mice learned similarly to discriminate the active hole from the inactive one. Precise analysis of behavior of homozygous Zif268 mutant mice revealed that these mice made significantly less nose-poking in the non-reinforced hole than wild type mice, particularly during the first training session (p<0.01, Bonferroni post-hoc test, see Fig. S1). As they also performed less correct responses, their ratio of reinforced responses over total responses was similar to that of wild type mice. After a few training sessions, poking in the non-reinforced hole became low in wild type mice and similar to the score observed in homozygous mutant mice (Fig. S1). Finally we did not detect any evidence of perseverative responses in the mutant mice. As the wild type mice, they performed the same number of pokes necessary to obtain food pellets, just above 1, 5 and 10 pokes under FR1, FR5 and FR10 schedules, respectively (Fig. S2).

**Figure 6:**
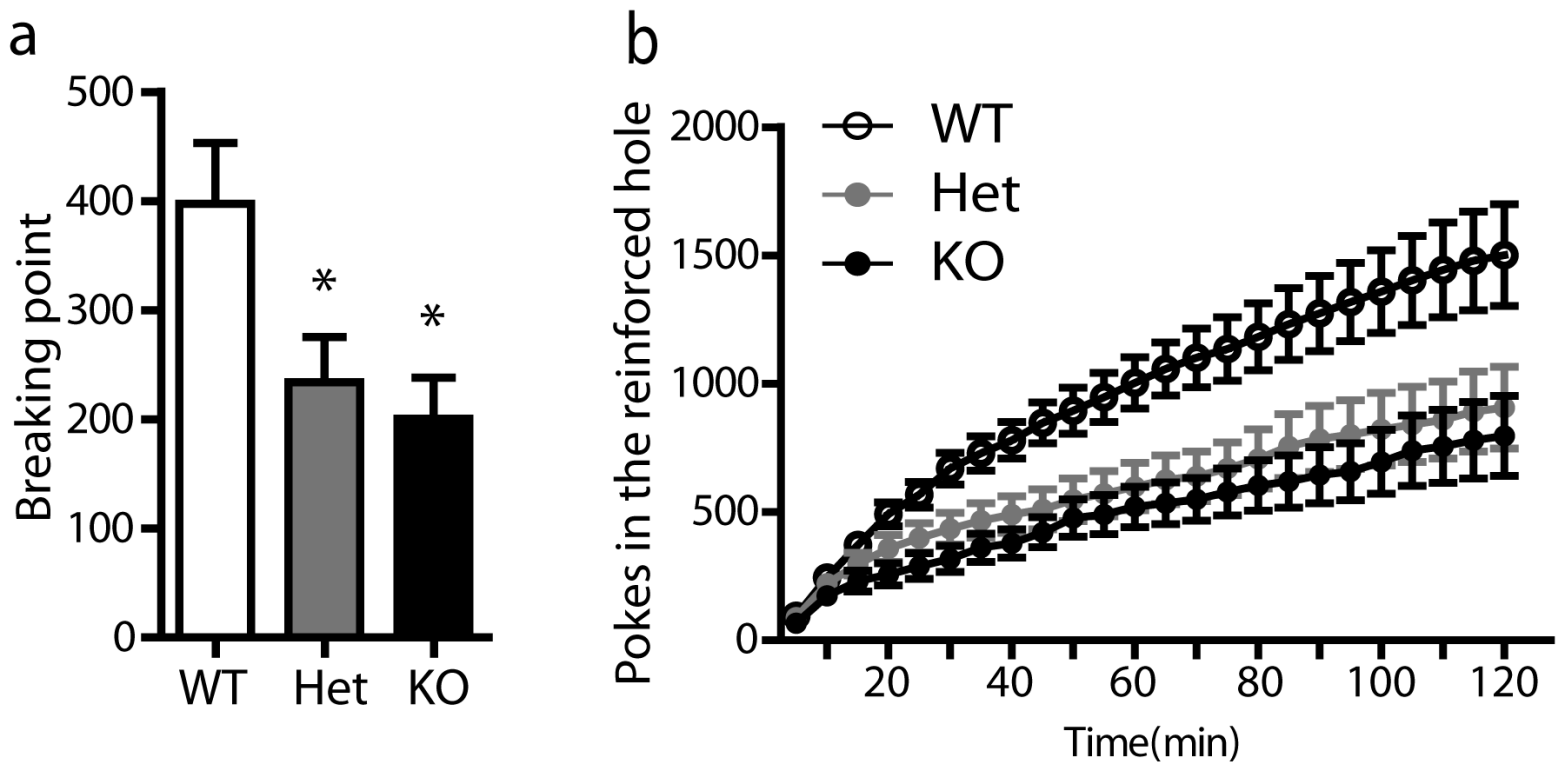
Operant behavior in Zif268 mutant mice under progressive ratio schedule. Groups of homozygous (**KO**, n = 11) and heterozygous (**Het**, n = 16) Zif268 mutant mice and their wild type (**WT**, n = 18) littermates were trained under progressive ratio (or PR) schedule. a) Breaking point measured during 2-h session under progressive ratio schedule. Both homozygous and heterozygous mutant mice show reduced breaking points when compared to wild type controls. The results (means ± SEM) are analyzed using one-way ANOVA (see the F value in Results). Post hoc comparison (Bonferroni test): * p<0.05. b) Cumulative number of nose-pokes into the reinforced orifice during progressive ratio schedule session (2 h). Both homozygous and heterozygous mutant mice show a reduced activity on the reinforced hole as compared to wild type controls. At 2 h, data were analyzed with one-way ANOVA (see the F value in Results).

We finally investigated the possibility that the mutation affected the motivation to work for food rewards. To test this, we subjected the three groups of animals to a 2-h test under progressive ratio schedule and we found that both heterozygous and homozygous mutant animals showed a significant marked reduction of breaking point (number of pokes for the last reward) compared to wild type mice (Fig. 6a, one-way ANOVA: F_(2,44)_ = 4.71, p<0.05). The time course of nose-pokes in the three genotypes showed a constant deficit of both homozygous and heterozygous mutant mice throughout the session (Fig. 6b) and a significant effect of genotype was detected on the cumulative number of nose-pokes into the reinforced orifice at 2 h (one-way ANOVA: F_(2,44)_ = 4.87, p<0.05). Under progressive ratio schedule, heterozygous Zif268 mutant mice appeared to be almost as affected as homozygous mice suggesting that the motivation to obtain food rewards is highly dependent on Zif268 influence.

## Discussion

In the present study, we explored the induction of Zif268 in various parts of the striatum during the first stages of training for an instrumental task and we determined its role in operant learning using Zif268-deficient mutant mice. We found that Zif268 was induced in the whole striatum following the first training session of learning, but this effect remained significant after the fifth session essentially in the lateral part of CPu, a striatal area involved in the formation of habits [6]. In the DM-CPu, a striatal region critical for the development of goal-directed behavior [6], Zif268 was not induced in yoked mice non-contingently receiving food reward after the first training session, suggesting that Zif268 induction in the trained animals was associated with some aspects of contingency. Zif268-deficient mice displayed altered performances in acquiring an instrumental task. We found particularly less reinforced pokes and less willingness to work for food rewards as evaluated with a progressive ratio schedule. By contrast, the possibility to discriminate between the reinforced and the non-reinforced holes was preserved in the mutant mice showing the persistence of associations of reward with discrete stimuli for driving instrumental response despite the marked alterations of the motivation to perform it.

### Localization of Zif268 induction in the striatum

We found that Zif268 was induced throughout the various areas of the striatum analyzed after the first session but remained elevated only in the lateral parts of the CPu after the fifth session. These results were consistent with previous experiments in rats showing that initial steps of instrumental learning increased Zif268 expression in the various parts of the striatum whereas after extensive overtraining Zif268 gene induction was reduced [36]. Interestingly, a similar restriction of the regional distribution of Zif268 induction was also observed following cocaine treatment, since cocaine produces Zif268 expression in the entire striatum after acute treatment, but only in dorsolateral parts of the striatum after repeated treatment [22], [37]. A similar progressive lateral transfer of expression has been also described for the immediate-early genes *Homer1* and *c-Fos*, during training for an instrumental task [36], [38]. It was found that learning-associated neuronal activity gradually shifts from the medial part of CPu to the lateral parts of CPu with progression of training [39]. Interestingly the lateral striatal regions, in which the Zif268 gene remained responsive after repeated sessions, are involved in the formation of habits that is thought to require longer periods of training [40]. Current models of information processing in the basal ganglia hypothesize that parallel loops connecting associative and sensorimotor cortices to basal ganglia contribute to the diverse basal ganglia functions [7], [41], [42]. The loop involving prelimbic prefrontal cortex and DM-CPu operates in the goal-directed behavior characterized by high dependence on the value of reward [43], [44], [45]. In contrast, the loop linking sensorimotor cortex and DL-CPu is critical for habitual actions in which stimulus-response associations dominate, with an insensitivity to reward devaluation [8]. In the course of acquisition of an instrumental task, goal-directed responding that prevails at the beginning is progressively replaced by habit-based reactions. Interaction between associative DM-CPu- and sensorimotor DL-CPu-based loops has been proposed to explain the transition from goal-directed to habit-driven behaviors. In this model, serial spiraling striato-nigro-striatal loops are organized in such a way that the medial striatal areas can modulate dopamine neurons innervating more lateral areas giving rise to a mechanism in which medial striatal area can control the activity in its adjacent lateral zone [46], [47], [48]. This effect would lead to a progressive involvement of more lateral parts of the striatum when the training increases and could be relevant to explain the lateral localization of Zif268 induction in the striatum after repeated training sessions.

### Zif268 induction in striatonigral and striatopallidal neurons

The striatal MSNs are divided into two populations projecting to the substantia nigra pars reticulata and lateral globus pallidus and giving rise to the direct and indirect pathways of the basal ganglia, respectively [49]. Despite high morphological similarities, MSNs of the direct and indirect pathways display marked differences in the expression of genes, particularly those encoding signaling molecules [50], [51]: a critical difference is the high expression of dopamine D1R in direct pathway neurons, contrasting with the high levels of dopamine D2R expression in indirect pathway neurons [32], [34]. Growing evidence indicates that the two populations of neurons are involved in different types of learning. It was shown that behavioral reinforcement is produced by specific stimulation of neuronal activity in the direct pathway and impaired by its inhibition [52], [53], [54]. Mirror effects were observed with conditioned aversion [53], [54], leading to the hypothesis that the direct pathway supports reinforcement learning whereas the indirect pathway controls punishment-induced aversion responses [54], [55]. After instrumental task training, we observed an induction of Zif268 in both neurons expressing and not expressing D1R, suggesting activity in both the direct and indirect pathways. In the course of the first training trials, the animals experience both successes and errors, and learn to facilitate correct responses and to inhibit incorrect ones. The induction of Zif268 in the two populations of MSNs could reflect plasticity processes consolidating correct responses and eliminating wrong ones. In contrast, *c-Fos* appeared to be significantly induced in the neurons of the indirect pathway suggesting its implication in processing inhibitory responses after the first training session. However, another study suggests that *c-Fos* is induced in D1R-expressing neurons in more trained animals after the transition from FR1 to FR5 schedule [38]. Interestingly, a recent study proposes that *c-Fos* induction in the striatum and others structures results from the attribution of incentive value to the reward-predictive cues [56].

### Zif268 induction in the DM-CPu

After the first training session, Zif268 levels in DM-CPu were significantly increased in the learning animals when compared to the yoked animals, not contingently receiving food rewards. It is highly unlikely that this response was simply due to motor activity associated with orifice exploration. Overtrained animals displayed no change in gene expression, while they performed many more nose-pokes compared with newly trained mice. Novelty or stress in a novel environment could not explain specific Zif268 induction in learning animals since yoked animals were exposed to exactly the same environmental stimuli. In addition, the activation was not related to the unexpected appearance of food or its intake, because yoked animals displayed striatal Zif268 levels similar to controls that did not receive any food rewards. At the end of this first training session, the learning animals displayed more nose-pokes into the cued orifice than yoked animals and began to discriminate the reinforced orifice from the non reinforced one. Experiencing and/or learning contingency, as indicated by these behavioral changes, could represent determinant factors leading to Zif268 induction.

### Role of Zif268 in instrumental learning

The functional importance of Zif268 induction in operant conditioning is supported by the impaired performance of Zif268-deficient mice in an instrumental task. However, these mice, in which the Zif268 gene is invalidated in all brain regions, cannot address the issue of the region specificity. It is possible that the Zif268 disruption in the striatum is responsible for the behavioral deficits reported here, given the important control exerted by striatum on operant conditioning [6], but it cannot be excluded that deficiencies in other brain regions, such as cortex, hippocampus or hypothalamus, also contribute to the phenotype. In the mutant mice, the behavior in the instrument task is characterized by an overall reduction in the number of pellets earned and nose-pokes in the active target. The deficit was more pronounced in the first sessions under FR1 and FR5 schedules and tended to decrease in the last sessions under FR10 schedule. By contrast, discrimination between reinforced and non-reinforced goals was not significantly altered in these mutant mice. Zif268-deficient mice were able to develop preference for rewarded goals with the same performance as the wild type mice, showing that they kept efficient ability to evaluate reward value of food. The fact that the mutant animals do not show alteration of food-conditioned place preference [22] leads to similar conclusions. Altogether, Zif268 mutant mice have a normal capacity to select rewarded responses in the operant conditioning model.

We observed that homozygous mutant mice performed fewer nose pokes in both the active and inactive holes during the first sessions, suggesting a possible deficit in exploratory behavior in these mice. However, this effect completely disappeared in the later sessions. In addition, previous studies reported that homozygous, heterozygous and wild type mice displayed similar spontaneous locomotor activities and habituation to novel environment [22], [35]. In the present study, we did not detect any reduction of the number of visits to the empty cup in the mutant mice. In conclusion all these data did not suggest a significant deficit of exploratory behavior of mutant mice.

Zif268-deficient mice displayed reduced initiation or execution of the adapted instrumental actions and important decrease of performance under progressive ratio schedule. This observed pattern of results, including less food earned, reduced responding on high-density (FR1, FR5) schedules with lower alteration on FR10 schedule, reduced responding on a progressive ratio schedule, and yet normal discrimination of reinforced from nonreinforced targets, could indicate an overall reduction of motivation for food (or appetite) in Zif268 mutant mice. This possibility is reinforced by the observation that the daily food intake by these mice was lower than in wild type. However, when we related the amount of food rewards earned during the training sessions to the daily food intake, the performance of Zif268 mutant was still impaired, suggesting that the phenotype was not completely explained by a non specific reduction of appetite. As indicated by the reduced nose-poking on a progressive ratio schedule, Zif268 mutant could in addition present a lower motivation to work for food rewards. The mechanisms underlying this motivation are unclear but Zif268 could contribute to consolidate memory traces associated with this function. The short-term memory is intact in Zif268 mutant mice as indicated by their normal responses in spatial working memory and short-term retentions for odors or objects. In contrast, long term memory in Zif268 mutant mice is severely impaired in several tasks including social transmission of food preference, object recognition, conditioned taste aversion, spatial navigation task in the water-maze and contextual fear conditioning [35], [57]. Zif268 is involved in the consolidation and reconsolidation of long term memories in various brain structures although the nature of these memories is very different from one structure to the other [19]. In the hippocampus, Zif268 contributes to the maintenance of late phase LTP and the consolidation and re-consolidation of hippocampal-dependent long-lasting memories [19], [35], [58]. In the NAc, Zif268 has been proposed to participate in the molecular mechanisms underlying the consolidation or re-consolidation of stimulus-drug associations that could determine drug-seeking behavior produced by drug-associated stimuli [59]. Because CPu is involved in processes necessary to attribute a motivational value to food-conditioned responses and produce responses in instrumental task [60], Zif268 in the CPu could contribute to consolidation or reconsolidation of memory traces in the context of instrumental learning. Alternatively, neural activity in the CPu has been shown to encode specific action sequences that are essential for operant conditioning [61]. A targeted mutation specifically disrupting learning of action sequences without affecting discrimination of action value was found to greatly impair the performance in instrumental task [61]. Similarly, Zif268 mutation may well alter the consolidation of sequential patterns of actions and, thereby, decrease the frequency of responses in the instrumental task despite the preserved discrimination and selection of rewarded goals. In conclusion, Zif268 appears to participate mainly in functions that regulate the intensity of work for food reward and less in those involved in the discrimination and selection of reinforced goals. It may contribute either to specific reward-related processes regulating motivation, or to mechanisms, such as learning of action sequences, that are important in the efficient execution of operant task.

## Supporting Information

Figure S1 **Number of nose-pokes in the non-reinforced target performed by Zif268 mutant mice in an instrumental task.** The number of nose-pokes in the non-reinforced target was measured in homozygous (KO, n = 11) and heterozygous (Het, n = 16) Zif268 mutant mice as well as in their wild type (WT, n = 18) littermates, across daily sessions with FR1, FR5 and FR10 schedule training. Data were analyzed by repeated-measures two-way ANOVA (within-subjects factor of Session and between-subjects factor of Genotype): FR1: Session: F_(4,168)_ = 17.6, p<0.001; Genotype: F_(2,42)_ = 5.17, p<0.01; interaction: F_(8,168)_ = 0.995, NS. FR5: Session: F_(4,168)_ = 3.76, p<0.01; Genotype: F_(2,42)_ = 0.644, NS; interaction: F_(8,168)_ = 0.787, NS. FR10: Session: F_(4,168)_ = 0.373, NS; Genotype: F_(2,42)_ = 0.714, NS; interaction: F_(8,168)_ = 1.53, NS. All the values are mean ± SEM. Overall two-way ANOVA results for genotype are indicated by: °°: p<0,01.
(TIF)

Figure S2 **Number of nose-pokes per reward performed by Zif268 mutant mice in an instrumental task.** The number of nose-pokes in the active target per reward was measured in homozygous (KO, n = 11) and heterozygous (Het, n = 16) Zif268 mutant mice as well as in their wild type (WT, n = 18) littermates, across daily sessions with FR1, FR5 and FR10 schedule training. Dashed line indicates the minimal number of pokes for pellet delivery. Data were analyzed by repeated-measures two-way ANOVA (within-subjects factor of Session and between-subjects factor of Genotype): FR1: Session: F_(4,168)_ = 5.65, p<0.001; Genotype: F_(2,42)_ = 0.222, NS; interaction: F_(8,168)_ = 0.995, NS. FR5: Session: F_(4,168)_ = 1.40, NS; Genotype: F_(2,42)_ = 0.73, NS; interaction: F_(8,168)_ = 1.45, NS. FR10: Session: F_(4,168)_ = 1.65, NS; Genotype: F_(2,42)_ = 0.26, NS; interaction: F_(8,168)_ = 1.46, NS. All the values are mean ± SEM.
(TIF)
