# Supplementary figures and images for: Role of the Plasticity-Associated Transcription Factor Zif268 in the Early Phase of Instrumental Learning
Source: PLoS One. 2014 Jan 23;9(1):e81868. doi: 10.1371/journal.pone.0081868 (PMC3900405; doi:10.1371/journal.pone.0081868)

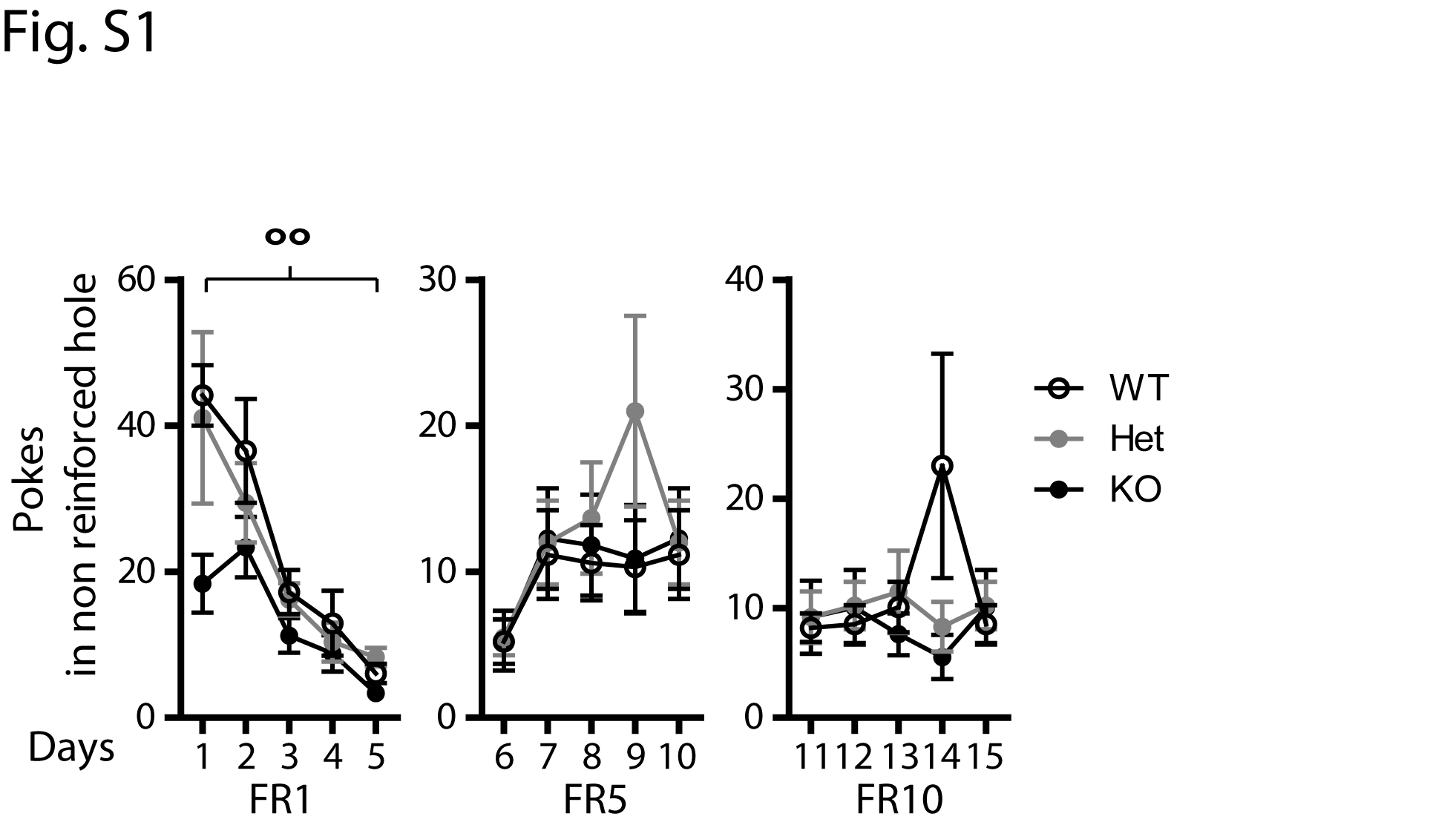

Supplement: Figure S1 — Number of nose-pokes in the non-reinforced target performed by Zif268 mutant mice in an instrumental task. The number of nose-pokes in the non-reinforced target was measured in homozygous (KO, n = 11) and heterozygous (Het, n = 16) Zif268 mutant mice as well as in their wild type (WT, n = 18) littermates, across daily sessions with FR1, FR5 and FR10 schedule training. Data were analyzed by repeated-measures two-way ANOVA (within-subjects factor of Session and between-subjects factor of Genotype): FR1: Session: F(4,168) = 17.6, p<0.001; Genotype: F(2,42) = 5.17, p<0.01; interaction: F(8,168) = 0.995, NS. FR5: Session: F(4,168) = 3.76, p<0.01; Genotype: F(2,42) = 0.644, NS; interaction: F(8,168) = 0.787, NS. FR10: Session: F(4,168) = 0.373, NS; Genotype: F(2,42) = 0.714, NS; interaction: F(8,168) = 1.53, NS. All the values are mean ± SEM. Overall two-way ANOVA results for genotype are indicated by: °°: p<0,01. (TIF) [file pone.0081868.s001.tif]

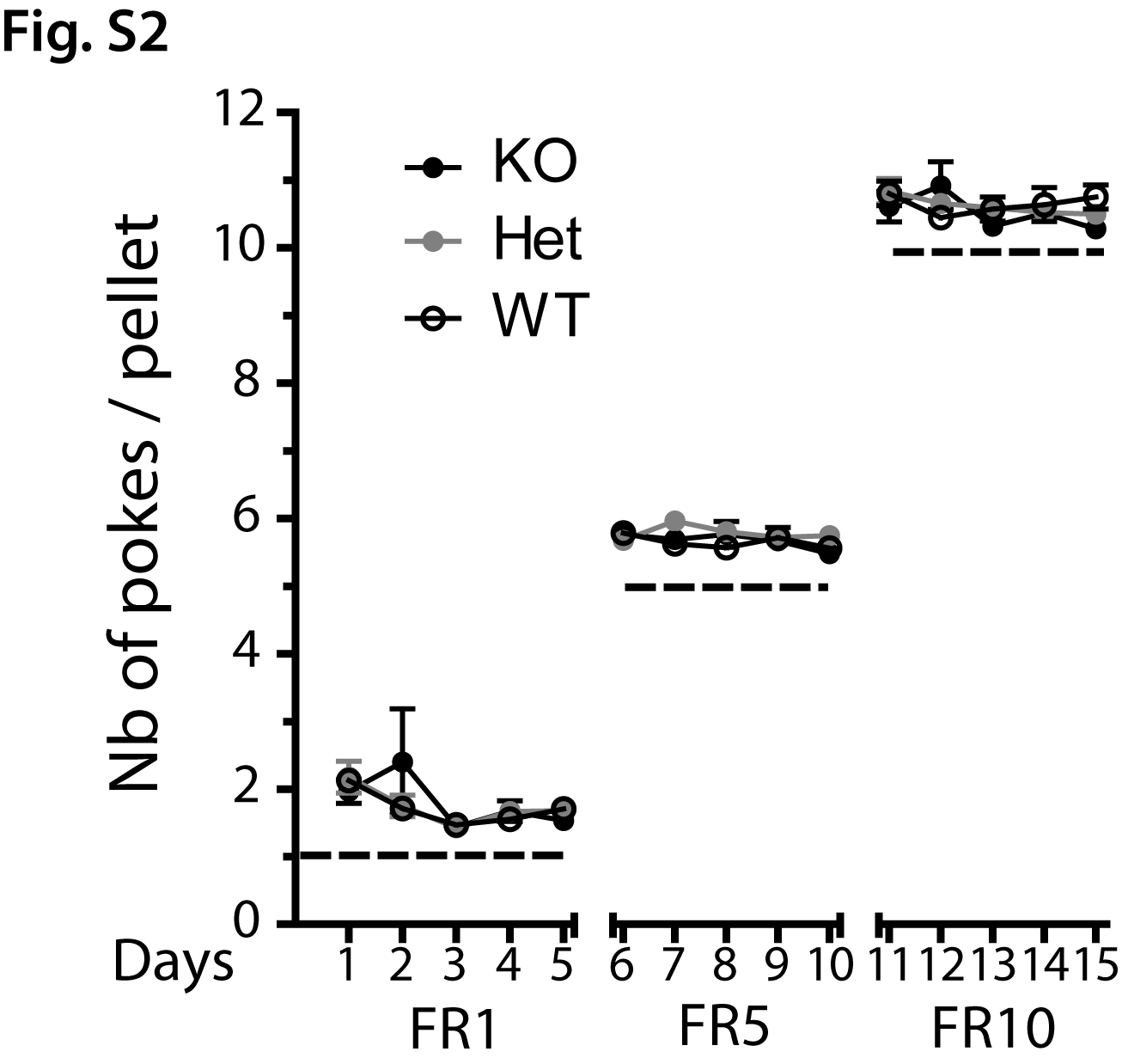

Supplement: Figure S2 — Number of nose-pokes per reward performed by Zif268 mutant mice in an instrumental task. The number of nose-pokes in the active target per reward was measured in homozygous (KO, n = 11) and heterozygous (Het, n = 16) Zif268 mutant mice as well as in their wild type (WT, n = 18) littermates, across daily sessions with FR1, FR5 and FR10 schedule training. Dashed line indicates the minimal number of pokes for pellet delivery. Data were analyzed by repeated-measures two-way ANOVA (within-subjects factor of Session and between-subjects factor of Genotype): FR1: Session: F(4,168) = 5.65, p<0.001; Genotype: F(2,42) = 0.222, NS; interaction: F(8,168) = 0.995, NS. FR5: Session: F(4,168) = 1.40, NS; Genotype: F(2,42) = 0.73, NS; interaction: F(8,168) = 1.45, NS. FR10: Session: F(4,168) = 1.65, NS; Genotype: F(2,42) = 0.26, NS; interaction: F(8,168) = 1.46, NS. All the values are mean ± SEM. (TIF) [file pone.0081868.s002.tif]
